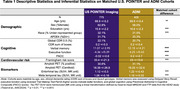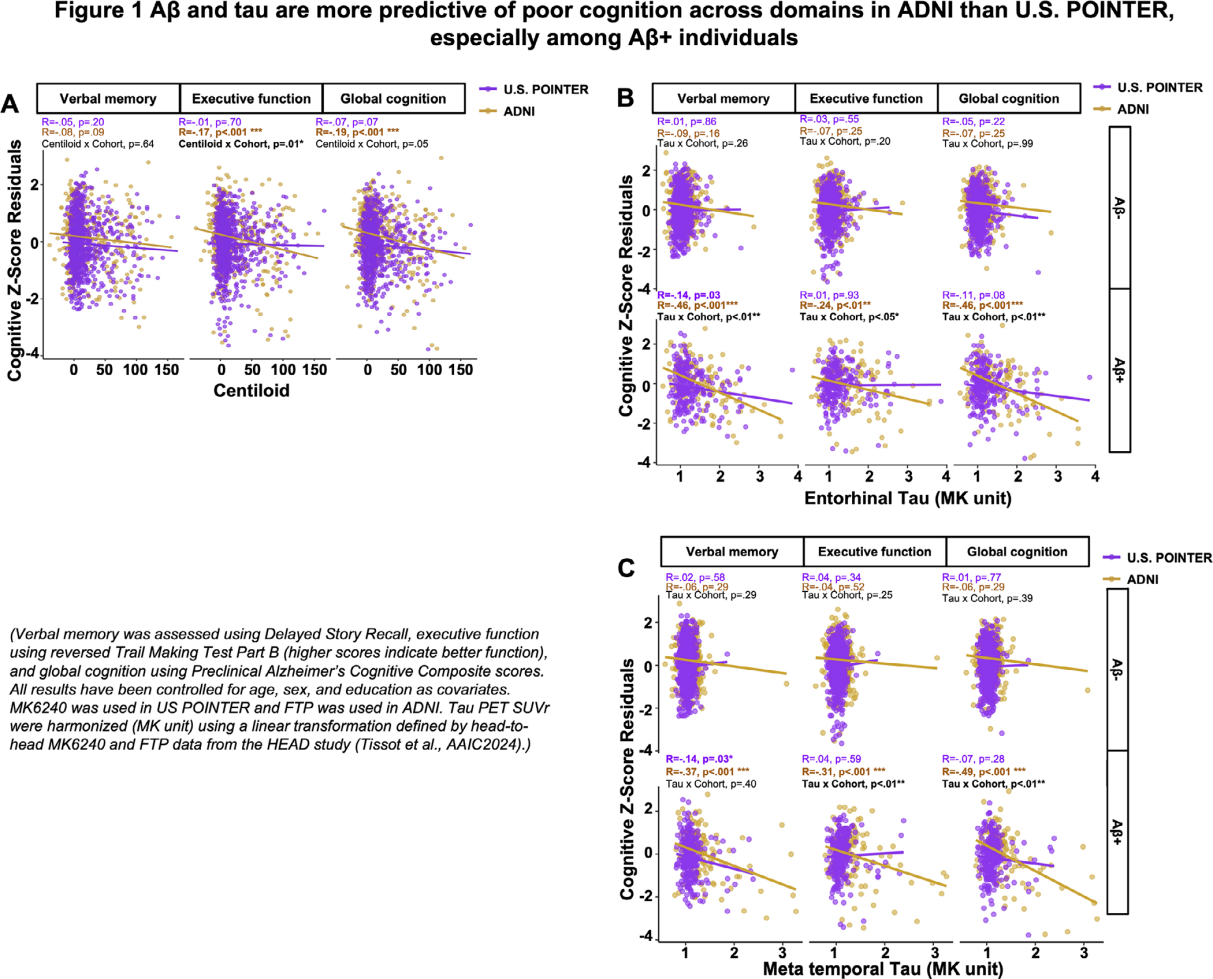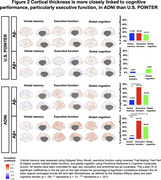# Does cohort heterogeneity affect relationships between AD biomarkers and cognition?

**DOI:** 10.1002/alz70856_100140

**Published:** 2025-12-25

**Authors:** Peiwei Liu, Jacinda Taggett, Theresa M. Harrison, Heather M Snyder, Charles Decarli, Prashanthi Vemuri, Danielle J. Harvey, Robert Koeppe, William J. Jagust, Laura D Baker, Susan M. Landau, the Alzheimer's Disease NeuroImaging Initiative and the US POINTER Study Group

**Affiliations:** ^1^ University of California, Berkeley, Berkeley, CA, USA; ^2^ Alzheimer's Association, Chicago, IL, USA; ^3^ Department of Neurology & Imaging of Dementia and Aging Laboratory, University of California Davis, Sacramento, CA, USA; ^4^ Department of Radiology, Mayo Clinic, Rochester, MN, USA; ^5^ University of California, Davis School of Medicine, Sacramento, CA, USA; ^6^ University of Michigan, Ann Arbor, MI, USA; ^7^ Wake Forest University School of Medicine, Winston‐Salem, NC, USA; ^8^ Neuroscience Department, University of California, Berkeley, Berkeley, CA, USA

## Abstract

**Background:**

Extensive evidence links cognition, tau, and neurodegeneration in Alzheimer's disease (AD). However, in U.S. POINTER imaging (a community‐recruited clinical trial cohort selected for high cardiovascular risk, ethnoracial diversity, and increased risk of cognitive decline and dementia) tau PET is more closely linked to cognition than in ADNI. Here, we further explore how ADNI and POINTER differ with respect to relationships between cross‐sectional AD biomarkers, cortical thickness, and different cognitive domains.

**Method:**

U.S. POINTER imaging (Baseline; *N* = 775; 68.9±5.2 years, 62.8% female) and ADNI (*N* = 405; 69.9±5.4 years, 58% female) were matched by age, sex, clinical status, and APOE4 genotype (Table 1). We examined associations between baseline AD biomarkers (Amyloid‐beta (Aβ) PET in centiloids, harmonized tau PET measures of MK6240 in POINTER and FTP in ADNI), regional cortical thickness, and verbal memory (Delayed Story Recall), executive function (Trail‐Making B), and global cognition (Preclinical Alzheimer's Cognitive Composite scores) across each cohort, controlling for age, sex, and education.

**Result:**

Aβ burden in ADNI was associated with worse executive function (*p* < .001) and global cognition (*p* < .001) but not memory, whereas in POINTER Aβ burden was not associated with any cognitive domain (Figure 1A). Among Aβ+ individuals, tau burden was associated with poorer performance across domains in ADNI, but only with poorer verbal memory in POINTER (Figures 1B&1C). Additionally, there were stronger positive associations between cortical thickness and executive function and global cognition compared to verbal memory, particularly in ADNI Aβ+ individuals even after adjusting for entorhinal tau (*p* < .01; Figure 2).

**Conclusion:**

Cognitive performance was linked to all biomarkers (Aβ, tau, cortical thickness) but these associations were consistently stronger in ADNI compared to POINTER, particularly in Aβ+ individuals. The stronger thickness‐cognition relationships in ADNI were not driven by tau, indicating that there is a tau‐independent role of thickness in explaining cognition. In ADNI, there were subtle differences between cognitive domains such that executive function was more closely linked than verbal memory to Aβ and cortical thickness, whereas verbal memory was linked to tau in both cohorts. Our study highlights that frequently‐reported AD biomarker associations may be attenuated in heterogeneous, community‐recruited cohorts.